# Identifying patients with metformin associated lactic acidosis in the emergency department

**DOI:** 10.1007/s11096-020-01069-2

**Published:** 2020-09-22

**Authors:** I. R. F. van Berlo-van de Laar, A. Gedik, E. van ‘t Riet, A. de Meijer, K. Taxis, F. G. A. Jansman

**Affiliations:** 1 Department of Clinical Pharmacy, Deventer Hospital, Nico Bolkesteinlaan 75, 7400 GC, P.O. Box 5001, 7416 SE Deventer, The Netherlands; 2 Department of Internal Medicine, Deventer Hospital, Nico Bolkesteinlaan 75, 7416 SE Deventer, The Netherlands; 3 Department of Research and Innovation, Deventer Hospital, Nico Bolkesteinlaan 75, 7416 SE Deventer, The Netherlands; 4 Department of Intensive Care, Deventer Hospital, Nico Bolkesteinlaan 75, 7416 SE Deventer, The Netherlands; 5 Unit of PharmacoTherapy, -Epidemiology &-Economics, Groningen Research Institute of Pharmacy (GRIP), University of Groningen, Antonius Deusinglaan 1, 9713 AV Groningen, The Netherlands

**Keywords:** Acidosis, Diagnosis, Lactic, Metformin, Renal insufficiency, Sepsis

## Abstract

*Background* Metformin associated lactic acidosis (MALA) is a serious adverse event with a high mortality rate of 30–50%. Early recognition of MALA and timely starting treatment may reduce its morbidity and mortality. *Objective* The aim of this study was to explore clinical parameters to identify patients with MALA in patients with suspected sepsis induced lactic acidosis in the emergency department ED. *Setting* A retrospective single centre study was conducted at the Deventer Teaching Hospital in the Netherlands. *Method* Patients with lactate concentration > 4.0 mmol/l admitted at the ED between 2010 and 2017 with suspected sepsis or confirmed MALA and referred to the Intensive Care Unit were included. Baseline characteristics (pH, lactate, creatinine and CRP) of MALA patients were compared with patients with suspected sepsis induced lactic acidosis. Creatinine and lactate concentration were selected as potential relevant parameters. *Main outcome measure* Sensitivity and specificity of the highest tertiles of the creatinine and the lactate concentrations separately, in combination, and both combined with metformin use, were calculated. *Results* Thirteen MALA and 90 suspected sepsis induced lactic acidosis patients were included. Lactate (14.7 vs 5.9 mmol/l, p < 0.01) and creatinine concentration (642 vs 174 μmol/l, p < 0.01) were significantly higher in the MALA group and arterial pH (7.04 vs 7.38, p < 0.01) and CRP (90 vs 185 mg/l, p < 0.01) were significantly lower. The combined parameters lactate ≥ 8.4 mmol/l, creatinine ≥ 256 μmol/l had a sensitivity of 85% and a specificity of 95% for identifying MALA in suspected sepsis induced lactic acidosis patients in the ED. When combined with metformin use the specificity increased to 99%. *Conclusion* When managing lactic acidosis in the ED the diagnosis MALA should be considered in patients with a creatinine concentration ≥ 256 μmol/l and lactate concentration ≥ 8.4 mmol/l.

## Impacts on practice

- Early recognition of metformin associated lactic acidosis (MALA) and timely starting treatment may reduce its morbidity and mortality.
- Clinicians should be alert to MALA in the emergency department, especially in patients with a creatinine concentration ≥ 256 μmol/l and lactate concentration ≥ 8.4 mmol/l.
- Metformin serum concentration measurement should be available 24 h a day for diagnosis and management of MALA.

## Introduction

Metformin is recommended as the treatment of choice in patients with type 2 diabetes mellitus because it decreases cardiovascular morbidity and mortality [1, 2]. Metformin associated lactic acidosis (MALA) is a serious adverse event with a high mortality rate of 30–50% [3]. The reported incidence of MALA varies from 0 to 138 per 100.000 patient years but this may be increasing the coming years due to the increase in the number of type 2 diabetes mellitus patients and the use of metformin [3–5]. In addition, the guidelines for prescribing metformin have been changed: metformin use is not contraindicated any more in patients with more severe renal failure (eGFR < 45 ml/min) [6].

The clinical symptoms of MALA and sepsis are similar, but the treatment is different. As in sepsis, MALA is often accompanied by organ dysfunction e.g. renal failure and hypotension [7]. Furthermore, in sepsis, lactate concentrations are frequently increased [8, 9]. Several studies suggest that starting timely treatment of MALA might reduce MALA-related morbidity and mortality [5, 10–15]. Identifying MALA patients in an early stage may also avoid unnecessary expensive and high risk diagnostic tests as e.g. CT-abdomen at the ED. Information about metformin use is not always known or fast available on admission at the ED. Measuring metformin serum concentrations can be helpful in diagnosing MALA but this is not routinely available in most hospitals [16–18].

In the treatment of MALA, extracorporeal treatment (ECTR) may be necessary to remove metformin, clear lactate and correct acid–base abnormalities [19]. The Extracorporeal Treatments in Poisoning Workgroup (EXTRIP) Group formulated recommendations for ECTR in the treatment of metformin poisoning [19, 20]. Clinical criteria to identify sepsis and septic shock are described by Singer et al. [21]. The management of the septic patient is described in the Surviving Sepsis Campaign International Guidelines 2016 [8]. Lactate is the most common serologic test used in the ED for risk stratification in suspected sepsis patients to optimize treatment but hyperlactatemia may be caused by conditions other than sepsis [8, 21, 22]. The differentiation between different origins of hyperlactatemia can be very difficult in clinical practice and there is a risk of misclassification [23]. In sepsis lactate is formed under hypoxic conditions and in MALA there is no evidence of a hypoxic condition [23]. The most widely accepted and established mechanism of hyperlactatemia and metabolic acidosis of metformin is partial inhibition of oxidative phosphorylation complex 1 of the mitochondrial electron transport chain. Another possible mechanism in which metformin may elevate plasma lactate levels is through inhibition of pyruvate carboxylase which results in both accelerated lactate production and reduced lactate metabolism [24, 25].

## Aim of the study

Early recognition of MALA and timely starting treatment may reduce morbidity and mortality. Therefore, the aim of this study was to explore clinical parameters that could be used to identify MALA patients in patients with suspected sepsis induced lactic acidosis in the ED intended for use in further research.

## Ethics approval

This study was performed after written consent of the Institutional Review Board and Board Director of the Deventer Teaching Hospital. Due to the retrospective nature of the study in which patient data were used anonymously, no informed consent of patients was needed.

## Methods

A retrospective single centre study was conducted at the Deventer Teaching Hospital in the Netherlands. Laboratory data were searched for patients admitted at the ED with lactate levels > 4.0 mmol/l between January 2010 and January 2017. Patients with the diagnosis suspected sepsis or a confirmed diagnosis of MALA and who were admitted at the ICU were included. Patients who were not admitted at the ICU, who had a diagnosis other than suspected sepsis or MALA, who died within 24 h after admittance and all patients with an intentional overdose with metformin were excluded.

The following data were extracted from the medical records: age, gender, Acute Physiology and Chronic Health Evaluation II (APACHE II), treatment (supportive care or extracorporeal treatment ECTR), mortality and laboratory results on admission: serum concentrations of creatinine, lactate, C-reactive protein (CRP) and metformin and blood pH. The APACHE II score is a common ICU scoring system to classify disease state and to predict mortality risk and prognosis.

The diagnosis suspected sepsis was extracted from the medical records and was based on quick Sequential Organ Failure Assessment (qSOFA) and or systemic inflammatory response syndrome (SIRS) criteria.

The included patients were divided into 2 groups: MALA and suspected sepsis induced lactic acidosis. MALA was confirmed by a pH < 7.35 and lactate concentration > 5.0 mmol/l and a metformin serum concentration > 5 mg/l, simultaneously measured at admission [3]. In the Deventer Teaching Hospital the metformin assay is routinely available 24 h a day. Results are available for clinical decisions within 4 h.

Baseline characteristics between groups were compared using the Mann–Whitney test because of non-normal distributed data. Based on clinical experience and previous studies, creatinine and lactate concentration were selected as potential diagnostic clinical parameters and were categorized into tertiles [4, 5, 7, 11–13, 15, 18, 26–29]. The sensitivity and specificity of the highest tertiles of these concentrations were calculated separately, in combination and both combined with metformin use respectively. The sensitivity was calculated by dividing the number of MALA patients with the specified clinical parameter(s) by the total number of MALA patients. The specificity was calculated by dividing the number of patients without MALA not having the specified clinical parameter(s) by the total number of patients without MALA.

In all tests, a p-value < 0.05 was considered statistically significant. Data analysis was performed with SPSS version 24.0.

## Results

In total 103 patients were included: 13 MALA patients and 90 suspected sepsis induced lactic acidosis patients. Patient characteristics of both groups are depicted in Table 1. There was no difference in age and APACHE II score between groups. The MALA group consisted of significantly more women compared to the suspected sepsis induced lactic acidosis group (85% versus 40%). Lactate and creatinine concentration were significantly higher in the MALA group and arterial pH and CRP were significantly lower. The treatment modality ECTR was more frequently used and mortality was higher in the MALA group. 26% of the suspected sepsis induced lactic acidosis patients used metformin but did not comply with the definition of MALA. Four patients in the MALA group had positive urine cultures but none of them had positive blood cultures.

**Table 1:** Patient characteristics

Parameter	Suspected sepsis induced lactic acidosis	MALA	Statistical Analysis (p-value)
N (%)	90 (87)	13 (13)	
Age (years) (median, percentiles 25–75)	70 (64–80)	72 (63–81)	0.74
Gender male (%)	54 (60)	2 (15)	< 0.01
Creatinine (μmol/l) (median, percentiles 25–75)	174 (118–258)	642 (260–801)	< 0.01
Lactate (mmol/l) (median, percentiles 25–75)	5.9 (4.8–8.3)	14.7 (11.0–22.5)	< 0.01
pH (median, percentiles 25–75)	7.38 (7.28–7.43)	7.04 (6.83–7.18)	< 0.01
CRP (mg/l) (median, percentiles 25–75)	185 (90–305)	90 (34–145)	< 0.01
Metformin use (%)	23 (26)	13 (100)	
Metformin (mg/l) (mean ± sd)	^a^	27.5 ± 15.3	
APACHE II^b^ (median, percentiles 25–75)	26.5 (21.0–32.3)	26.0 (23.0–33.0)	0.53
ECTR yes (%)	9 (10)	10 (77)	< 0.01
Mortality (%)	19 (21)	6 (46)	0.049

*MALA* metformin associated lactic acidosis, *CRP* C-reactive protein, *APACHE* Acute Physiology and Chronic Health Evaluation II, *ECTR* extracorporeal treatment

^a^Metformin serum concentration in metformin users is not measured or below 5 mg/l

^b^APACHE II scores and mortality prediction as percentage death rate:

0–4 ≅ 1–4%  15–19 ≅ 12–24%  30–34 ≅ 75%

5–9 ≅ 3–8%  20–24 ≅ 30–40%   > 34 ≅ 85%

10–14 ≅ 7–15%  25–29 ≅ 35–55%

Lactate and creatinine concentration were selected as potential relevant clinical parameters.

**Fig. 1:**
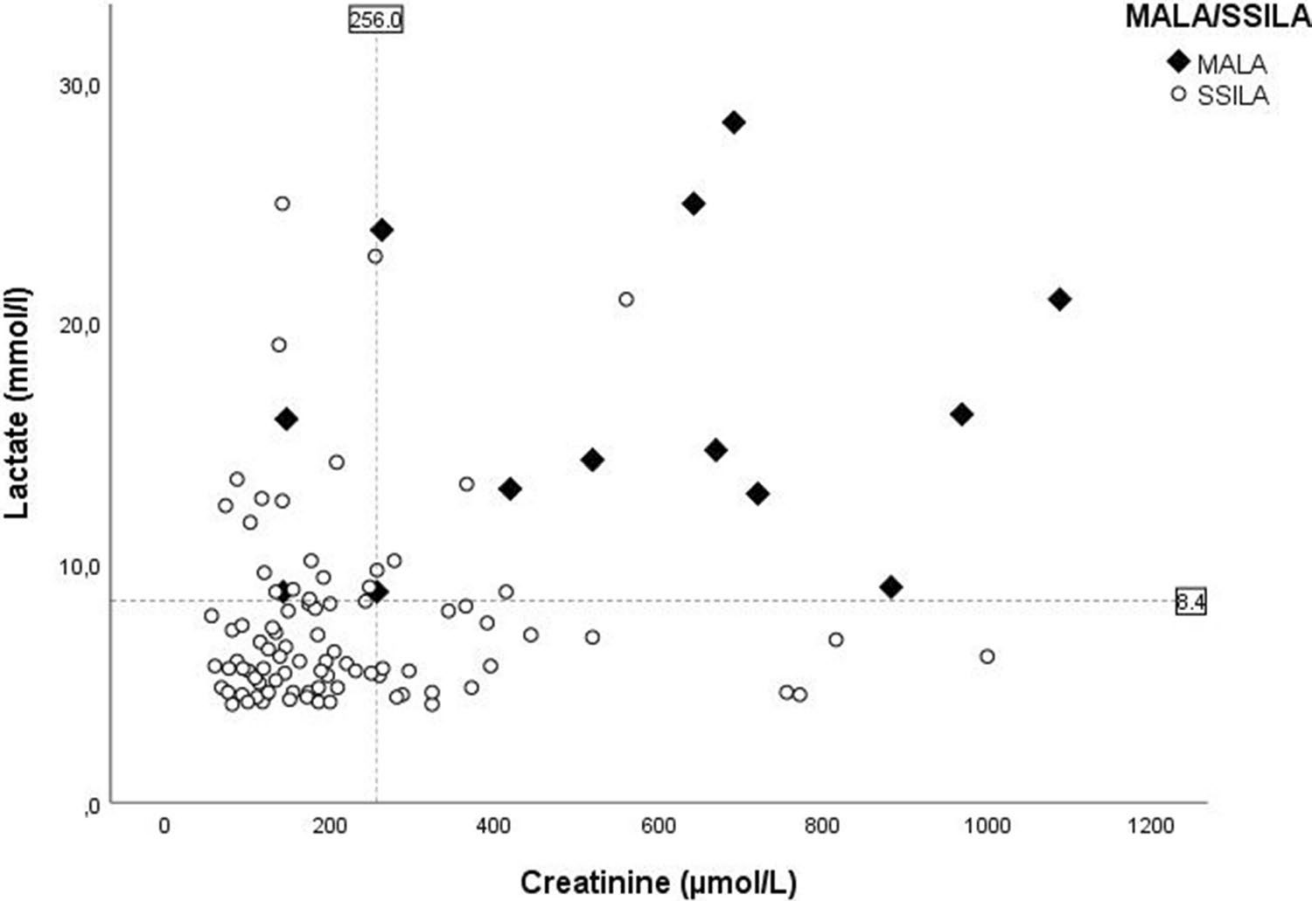
Scatterplot of creatinine and lactate concentrations from the MALA and suspected sepsis induced lactic acidosis patients. Dotted lines present the highest tertiles lactate and creatinine concentration. *SSILA* suspected sepsis induced lactic acidosis

Lactate concentration was categorized into tertiles resulting in the groups: ≤ 5.5 mmol/l, 5.6–8.3 mmol/l and ≥ 8.4 mmol/l. The creatinine concentration tertiles resulted in the groups: ≤ 142 μmol/l, 143–255 μmol/l and ≥ 256 μmol/l. A scatterplot in Fig. 1 shows the relationship between lactate and creatinine concentrations of all patients in relation to the highest tertiles.

The sensitivity and specificity of the highest tertiles of lactate and creatinine concentration are depicted in Table 2. Sensitivity and specificity are presented in relation to lactate and creatinine concentration separately, in combination and both in combination with metformin use. Among these, the combined parameters lactate ≥ 8.4 mmol/l, creatinine ≥ 256 μmol/l and metformin use showed the highest combination of sensitivity (85%) and specificity (99%) in identifying MALA patients from patients with suspected sepsis induced lactic acidosis in the ED.

**Table 2:** Sensitivity and specificity of parameters for identifying MALA patients in patients with suspected sepsis induced lactic acidosis

Clinical parameter	MALA N = 13	Patients without MALA N = 90
Lactate ≥ 8.4 mmol/l (N)	13	21
Lactate < 8.4 mmol/l (N)	0	69
**Sensitivity (%)/specificity (%)**	**100**	**76.6**
Creatinine ≥ 256 μmol/l (N)	11	23
Creatinine < 256 μmol/l (N)	2	67
**Sensitivity (%)/pecificity (%)**	**84.6**	**74.4**
Metformin use (yes)	13	23
Metformin use (no)	0	67
**Sensitivity (%)/pecificity (%)**	**100**	**74.4**
Lactate ≥ 8.4 mmol/l and creatinine ≥ 256 μmol (N)	11	5
Lactate < 8.4 mmol/l and/or creatinine < 256 μmol (N)	2	85
**Sensitivity (%)/specificity (%)**	**84.6**	**94.4**
Lactate ≥ 8.4 mmol/l and creatinine ≥ 256 μmol and metformin use (yes) (N)	11	1
Lactate < 8.4 mmol/l and/or creatinine < 256 μmol and/or metformin use (no) (N)	2	89
**Sensitivity (%)/specificity (%)**	**84.6**	**99.0**

*MALA* metformin associated lactic acidosis

## Discussion

The aim of this study was to explore clinical parameters to identify MALA patients in patients with suspected sepsis induced lactic acidosis in the ED. This is important because early recognition of MALA may improve treatment and reduce morbidity and mortality. The results show that the combined parameters lactate ≥ 8.4 mmol/l and creatinine ≥ 256 μmol/l have a sensitivity and specificity of 85% and 95% respectively for identifying MALA in suspected sepsis induced lactic acidosis in the ED. The specificity is increased to 99% when combining this with metformin use.

The MALA group in this study had more severe metabolic acidosis (higher lactate and lower pH) and more severe renal dysfunction (higher creatinine concentration) compared to the suspected sepsis induced lactic acidosis group. The clinical parameters (lactate, pH, creatinine, metformin serum concentration) of the MALA group in this study are in line with previous literature [4, 5, 7, 11–13, 15, 18, 26–29]. To our knowledge, this is the first study comparing MALA and suspected sepsis induced lactic acidosis patients. Three related studies were found [7, 10, 22]. Semely et al. [10] have shown that an early MALA diagnosis procedure in all patients with metformin admitted at the ED tends to decrease mortality, especially for serious MALA cases. Friesecke et al. [7] have compared MALA patients with patients with lactic acidosis of other origin (LAOO). They found, as in our study, a significantly higher lactate concentration and significantly lower blood pH in MALA patients. Green et al. [22] have shown in adult ED patients with suspected sepsis that metformin users had slightly higher lactate concentrations and hyperlactatemia but this was not associated with a higher mortality risk.

If MALA was not considered as a cause of the hyperlactatemia in this study, 13% of the patients would have received sepsis treatment and adequate MALA treatment was possibly started too late or not at all leading to a higher mortality. The mortality in our MALA group of 46% is in accordance with results presented in previous literature, i.e. 30–50% [5, 19]. In addition, the measured mean APACHE II scores of 26.5 matches the mortality in this study. APACHE II scores of 25–29 are associated with an approximated in-hospital mortality of 35–55% [30]. However, the mortality in the sepsis group of 21% is, relatively low compared to literature and the measured APACHE II score of 26.0 [8, 30]. Not all our sepsis patients had a confirmed sepsis diagnosis, which could be an explanation of the low mortality seen in this group. Patients were included with the diagnosis suspected sepsis based on SIRS or qSOFA scores in the medical files. We did not evaluate if the diagnosis sepsis of these patients was confirmed with microbiological data or otherwise because this study concerned the prediction of the identification of MALA patients in patients with suspected sepsis induced lactic acidosis in the ED.

We selected lactate and creatinine as potential relevant parameters to identify MALA patients in patients with suspected sepsis induced lactic acidosis in the ED. CRP was significantly lower in the MALA patients but adding CRP did not improve the predictive value (data not shown). We chose not to select pH as a relevant parameter because pH is also dependent of carbon dioxide.

A sensitivity of 85% is not optimal and a higher sensitivity is desirable. In this study, 2 out of 13 MALA patients are not identified using this approach. Both patients had a lactate concentration exceeding 8.4 mmol/l but their creatinine concentration was less than 256 μmol/l. MALA is often preceded by gastrointestinal symptoms e.g. nausea, vomiting and diarrhoea prior to admittance [4, 5, 7, 26]. The gastrointestinal problems lead to acute renal failure which subsequently leads to metformin accumulation causing lactic acidosis. Possibly, by taking gastrointestinal symptoms prior to admission into account, sensitivity could be improved.

The specificity of the combined parameters lactate and creatinine concentration is 95%. In Fig. 1 is shown that four sepsis patients have a lactate ≥ 8.4 mmol/l and a creatinine ≥ 256 μmol/l and are therefore falsely identified as MALA patients using these two parameters. These patients did not use metformin and the specificity can be increased to 99% by adding metformin use as clinical parameter. However, metformin use may not always be directly available at the ED.

A strength of this study is that we defined MALA based on simultaneously measurement of lactate, pH and metformin serum concentration at admission. Lalau et al. [3] identified the lack of these combined data as one of the methodological flaws in most studies about MALA. Another strength is our selection of MALA patients. To minimize the discussion if metformin or comorbidity is causing lactic acidosis in patients using metformin we added the criterion metformin serum concentration > 5 mg/l to the generally accepted criteria of MALA pH < 7.35 and lactate > 5 mmol/l as suggested by Lalau et al. [3].

A limitation of the study is that comorbidity was not extracted separately from the medical records because this was diverse in the study population and difficult to quantify and to extract form the electronic patient files. However, we did extract the APACHE II scores, which is a common ICU scoring system for classifying disease state and predicting mortality and prognosis. There was no significant difference in APACHE II scores between the MALA and the suspected sepsis induced lactic acidosis group, reflecting comparable disease states and mortality risks. Another limitation of our study regards to the retrospective single centre study design. In addition, the number of MALA patients (N = 13) was very low which makes drawing conclusions on clinical implications from this study difficult. Because of the relatively low patient number, it was also not possible to perform multivariate regression analysis, which is the method of first choice in prediction research, so we had to calculate the sensitivity and specificity of the selected parameters manually. With multivariate regression analysis, it is possible to estimate statistically significant relations between multiple independent clinical variables and outcome simultaneously. This would have made the results more accurate and robust. Furthermore, selection bias cannot be excluded in our study. We selected patients based on lactate concentrations > 4.0 mmol/l. There is no hard criterion defined for the lactate concentration to identify sepsis or septic shock. However, lactate levels exceeding 4 mmol/l correlate highly with underlying pathology and increased morbidity [22, 23, 31]. Therefore we selected patients with lactate > 4.0 mmol/l. However, patients with suspected sepsis and lactate < 4.0 mmol/l could have been missed. This also counts for MALA patients with no measured metformin concentrations. Finally, we divided the patients into two groups, i.e. MALA and suspected sepsis induced lactic acidosis based on the inclusion and exclusion criteria. However, possibly, there were combined diagnoses and other comorbidity contributing to hyperlactatemia. 26% of the sepsis group used metformin but did not comply with the definition of MALA. Four patients in the MALA group had positive urine cultures but none of them had positive blood cultures.

Our findings are of important clinical relevance because of the high mortality of MALA and the necessity of starting the right treatment immediately. The number of diabetes mellitus type 2 patients will increase the coming years and so is the use of metformin, thereby probably increasing the incidence of MALA [5]. Clinical symptoms of MALA and sepsis are similar and metformin serum concentration measurement is not routinely available in most hospitals. In addition, sepsis confirmation by microbiologically testing takes days. In the treatment of MALA, ECTR is often necessary to remove metformin, clear lactate and correct acid–base abnormalities [19]. Therefore, a simple diagnostic approach identifying MALA patients in patients with suspected sepsis induced lactic acidosis in the ED is of high clinical relevance. Lactate and creatinine are standard laboratory tests which are performed immediately at the ED when sepsis is suspected [8, 14, 22, 23, 31]. This study presents specific readily available clinical parameters in order to identify MALA patients, which gives direction in starting fast the right treatment, possibly leading to a better outcome. However, further research is necessary to improve the sensitivity of this approach and to validate the distinctive value in a prospective, multicentre design.

For clinical practice, we recommend that clinicians be alert to MALA in the ED when patients are admitted with severe lactic acidosis and renal failure. Knowledge of the metformin serum concentrations may be a valuable additional parameter for the diagnosis and management of MALA, despite the uncertainty concerning the prognostic value and the lack of a specific threshold for metformin to initiate ECTR [16–18]. Therefore, we recommend clinical pharmacists to organise access to a 24 h available metformin assay for hospitals treating MALA patients. Also, pharmacists can play an important role in preventing MALA by performing adequate medication surveillance in patients using metformin and having renal failure and educating patients to temporarily stop metformin when they have symptoms of diarrhoea and vomiting [4, 26].

## Conclusion

In case of lactic acidosis in the ED, besides sepsis, the diagnosis MALA should be considered in patients with a creatinine concentration ≥ 256 μmol/l and lactate concentration ≥ 8.4 mmol/l. The sensitivity and specificity of these parameters combined with metformin use to identify MALA patients in patients with suspected sepsis induced lactic acidosis were 85% and 99% respectively. As early recognition of MALA is essential for initiating the appropriate treatment in order to reduce morbidity and mortality and to avoid expensive and high risk diagnostic tests, these parameters should be considered in managing lactic acidosis in the ED. A prospective, multicentre design validating the distinctiveness of these set of parameters is recommended to confirm these results.
